# Breaking the silence—role of MucR as a virulence determinant in *Brucella*

**DOI:** 10.1128/jb.00170-25

**Published:** 2025-09-18

**Authors:** Ian S. Barton, Connor B. Cribb, Beatriz Tartilán-Choya, Graham J. Bitzer, Jodi Ogle, Neil Garza Hernandez, Daniel W. Martin, Nieves Vizcaíno, Xindan Wang, Ilaria Baglivo, R. Martin Roop

**Affiliations:** 1 Department of Microbiology and Immunology, Brody School of Medicine, East Carolina University 12278, Greenville, North Carolina, USA; 2 Department of Microbiology and Genetics, University of Salamanca 16779, Salamanca, Spain; 3 Department of Biology, East Carolina University 3627, Greenville, North Carolina, USA; 4 Department of Biology, Indiana University 1772, Bloomington, Indiana, USA; 5 Department of Environmental, Biological and Pharmaceutical Sciences and Technologies, University of Campania “Luigi Vanvitelli”, Caserta, Italy; Philipps-Universitat Marburg Fachbereich Biologie, Marburg, Germany

**Keywords:** *Brucella*, MucR, H-NS-like protein, nucleoid-associated protein, virulence

## Abstract

The Zn finger protein MucR is an H-NS-like protein that serves as a gene silencer and nucleoid-structuring protein in the α-proteobacteria. MucR is also an essential virulence determinant in *Brucella*, where it directly and indirectly controls the expression of many genes required for the virulence of these bacteria in their mammalian hosts, including those encoding the Type IV secretion system and its effectors, the autotransporter adhesins BtaE and BmaC, and the quorum sensing regulators VjbR and BabR. Experimental evidence suggests that one of the primary functions of the *Brucella* MucR is to ensure that virulence genes are only expressed when their corresponding gene products provide fitness benefits to these bacteria during their infectious lifecycle, and we propose that this function is central to the well-established role of MucR as a virulence determinant.

## MucR IS AN H-NS-LIKE GLOBAL REGULATOR IN THE α-PROTEOBACTERIA

**Fig 1:**
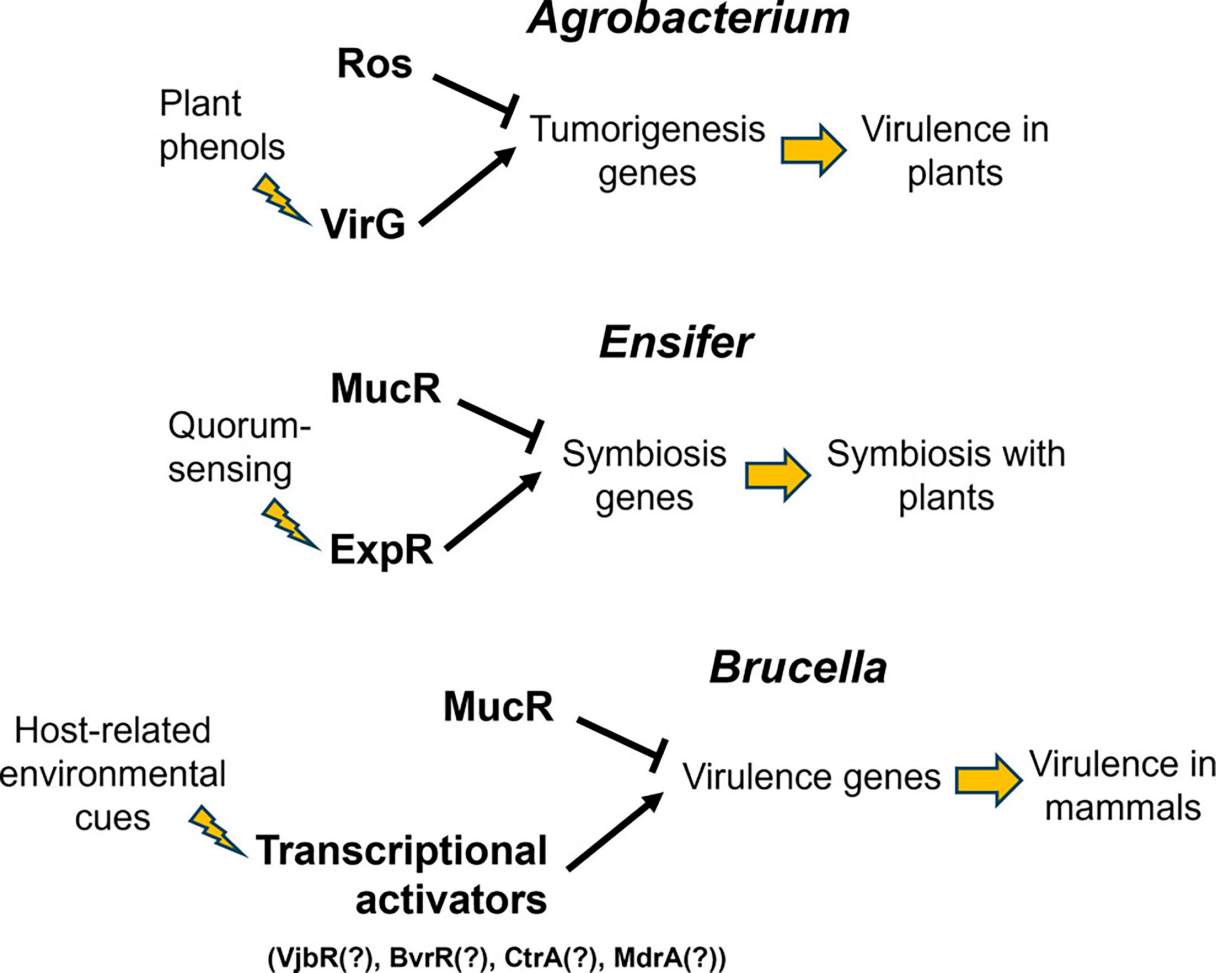
Proposed roles of MucR homologs in coordinating the proper temporal expression of host-interaction genes in *Agrobacterium tumefaciens* (13), *Ensifer* (formerly *Sinorhizobium*) *meliloti* (5), and *Brucella* (11).

 MucR, also known as Ros or RosR, is a C_2_H_2_-type Zn finger protein that serves as a global regulator in the α-proteobacteria (1, 2). The gene encoding MucR was originally discovered during genetic studies aimed at identifying genes linked to the regulation of virulence traits in *Agrobacterium* (3) and exopolysaccharide biosynthesis in *Rhizobium* (now *Ensifer*) (4). The “*ros*” and “*mucR*” designations are based on the “rough outer surface” and “mucoid” colony phenotypes displayed by the *Agrobacterium tumefaciens ros* and *Ensifer meliloti mucR* mutants, respectively. MucR homologs control the expression of extensive regulons often encompassing hundreds of genes in many of the α-proteobacteria (5–10). Notably, some of these genes play critical roles in the symbiotic and pathogenic interactions of these bacteria with plants and animals. Despite its well-established role as a global regulator, the precise molecular mechanisms by which MucR exerts its regulatory functions remained unclear for many years. But recent studies have shown that MucR is a nucleoid-structuring protein that functions in a manner analogous to the Histone-like Nucleoid Structuring (H-NS) and H-NS-like proteins described in other bacteria (11). Specifically, MucR binds to low-consensus AT-rich regions across the bacterial genome, and its capacity to form higher-order oligomers folding in an unusual circular structure allows it to compact and structure the nucleoid and serve as a global transcriptional repressor or “gene silencer” (2, 11, 12). The gene silencing function of MucR has been proposed to work in concert with antagonistic “counter-silencers” to coordinate the proper temporal expression of cell cycle, symbiosis, and virulence genes in the α-proteobacteria (5, 8, 13) (Fig. 1) and protect these bacteria from the potentially toxic effects of uncontrolled expression of foreign genes acquired by horizontal gene transfer (HGT) (14).

## MucR IS AN ESSENTIAL VIRULENCE DETERMINANT IN *BRUCELLA*

 *Brucella* strains are significant causes of abortion and infertility in food animals and sources of zoonotic infections worldwide (15). A link between MucR and *Brucella* virulence was first discovered during a screen of transposon mutants derived from *Brucella melitensis* 16M selected for attenuation in the J774.A1 murine macrophage-like cell line (16). Subsequent studies in several laboratories confirmed that *B. melitensis mucR* mutants are highly attenuated in cultured mammalian cells and mice (7, 16–18) and showed that *Brucella abortus*, *Brucella canis*, and *Brucella ovis mucR* mutants are likewise attenuated in these experimental models (6, 19, 20).

 The nucleoid-structuring function of MucR allows this protein to have both direct and indirect impacts on gene expression by altering global DNA topology (11) and directly influencing the interactions of RNA polymerase and transcriptional regulators with genes. Transcriptomics and proteomics indicate that over 400 *Brucella* genes are directly or indirectly regulated by MucR (6, 7, 18–20), and ChIP-seq analysis in *B. abortus* 2308 suggests that this number may be even greater depending upon the environmental conditions under which gene expression is being evaluated (11). Many MucR-regulated genes are involved in basic physiology and metabolism, and *Brucella mucR* mutants typically display pleiotropic phenotypes when they are cultivated *in vitro*, including altered outer membrane properties, growth defects and a reduced capacity to respond to multiple environmental stresses including oxidative stress, acidic pH, and Fe deprivation. These generalized fitness defects almost certainly contribute to the attenuation exhibited in *Brucella mucR* mutants by compromising their ability to overcome the harsh environmental conditions within the intracellular environment of their mammalian hosts (21). However, as pointed out below, evidence suggests that MucR also plays very specialized roles in *Brucella* virulence.

## MucR AND “COUNTER-SILENCERS” CONTROL VIRULENCE GENE EXPRESSION IN *BRUCELLA*

 Some virulence determinants are only beneficial to bacterial pathogens during specific stages of their infectious life cycle. Adhesins, for example, allow bacteria to attach to and/or enter mammalian cells and often impart specificity for certain host tissues (22). Effectors secreted by Type IV secretion systems, on the other hand, typically exert their direct impacts during the intracellular replication of bacteria in host cells (23). Thus, it is not surprising that uncontrolled expression of certain virulence genes in bacterial pathogens has been shown to have a detrimental effect on the infectious process and lead to attenuation (24).

 One of the major functions of the H-NS and H-NS-like gene silencers in bacterial pathogens is to prevent the uncontrolled and gratuitous expression of virulence genes (25). Transcriptional activators known as counter-silencers play an equally important role in ensuring the proper temporal expression of these genes by responding to host-specific environmental cues and overcoming this gene silencing. The concerted activities of these antagonistic gene silencer/counter-silencer pairs allow pathogens to ensure that genes encoding specific virulence determinants are selectively expressed at stages of the infectious process when they confer a fitness benefit (26, 27).

 As noted in a previous section, MucR has been proposed to function as an H-NS-like gene silencer and work in concert with antagonistic transcriptional activators to coordinate the proper temporal expression of virulence genes in *A. tumefaciens* (13) and symbiosis genes in *E. meliloti* (5). Recent evidence indicates that MucR plays the same regulatory role in *Brucella* (Fig. 1). Table 1 lists the *Brucella* virulence genes for which there is evidence suggesting co-regulation by MucR and specific transcriptional activators. Notably, two of these transcriptional activators, VjbR and BvrR, control gene expression in response to host-specific environmental stimuli. VjbR is the predominant quorum-sensing regulator in *Brucella* (28–30) and BvrR and the corresponding sensor BvrS regulate gene expression in response to the acidic pH and nutrient deprivation encountered in the intracellular environment of mammalian cells (31). CtrA is also notable because it is the master regulator of the cell cycle in *Brucella* (32) and *Brucella* cells arrested in phase G1 of the cell cycle are the most infectious for host cells (33). Similarly, NolR, MdrA, and HutC have also been linked to the activation of important *Brucella* virulence genes (34–37).

**TABLE 1:** *Brucella* virulence genes that are potential targets of coordinated regulation by MucR and counter-silencers

Gene	Possible counter-silencer(s)	Proposed function	References
*btaE*	VjbR, MdrA, HutC	Adhesin	(11, 38, 39)
*bmaC*	CtrA	Adhesin	(11, 32)
*omp25d*	NolR, EssR	Adhesin^a^	(11, 37, 40)
*virB1*	VjbR, BvrR	T4SS	(11, 39, 41, 42)
*virB2*	VjbR	T4SS	(11, 39, 41)
*vceC*	VjbR	T4SS effector	(11, 39, 41)
*btpB*	VjbR, BvrR	T4SS effector	(11, 39)
*asp24*	VjbR	Acid shock protein	(11, 39)
*sodC*	CtrA	Cu/Zn SOD	(11, 32)
*ba14k*	VjbR, CtrA	Unknown	(11, 32, 39)
*fliC*	CtrA	Flagellin	(11, 32)
*vjbR*	BvrR	Transcriptional regulator	(11, 42)
*ftcR*	VjbR, CtrA	Transcriptional regulator	(11, 32, 39)
*lovhK*	VjbR, CtrA	Transcriptional regulator	(11, 32, 39)

^a^ May have other functions across *Brucella* species including membrane homeostasis.

## ADHESINS

**Fig 2:**
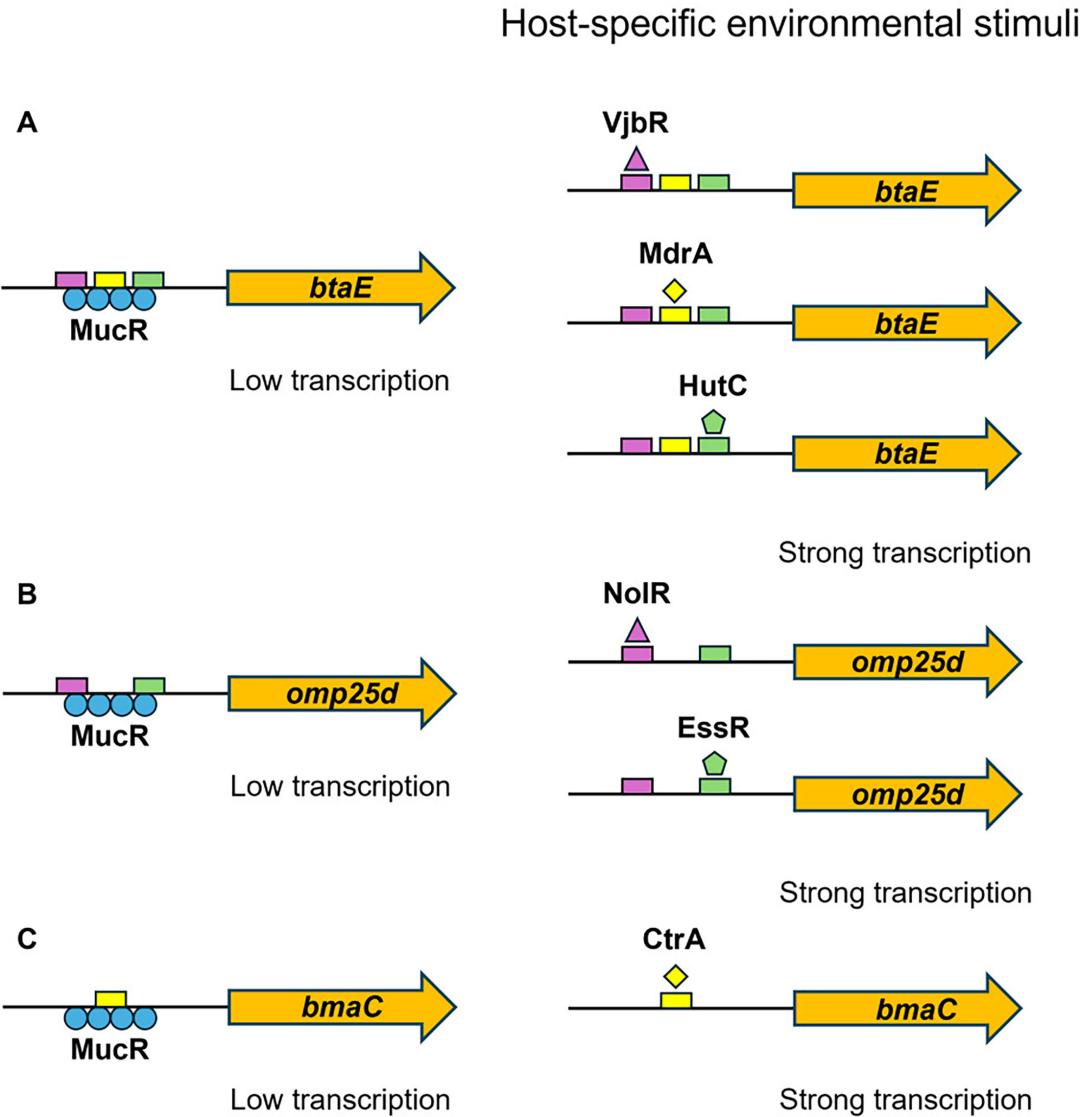
Proposed roles of MucR and counter-silencers in regulating the proper temporal expression of the genes encoding BtaE (**A**), Omp25d (**B**), and BmaC (**C**) in *Brucella*. Under non-permissive conditions, MucR represses transcription of these genes (left) until host-specific environmental stimuli activate counter-silencers that displace MucR and induce expression (right).

 The best experimental evidence for the existence of MucR/counter-silencer pairs coordinating virulence gene expression in *Brucella* comes from ongoing studies of MucR regulation of the genes encoding BtaE and Omp25d. BtaE is a polar autotransporter adhesin that plays an important role in the attachment of *B. suis* and *B. abortus* strains to epithelial cells (22, 38). Omp25d is an outer membrane protein that is required for full virulence of *B. suis* in mice and may be involved in membrane homeostasis (37), but also affects the capacity of *B. ovis* to attach to and invade epithelial cells and macrophages (43), suggesting that Omp25d may play multiple roles in virulence across *Brucella* spp. Both proteins are produced at very low levels in *Brucella* cells during routine *in vitro* cultivation, suggesting that the corresponding genes are tightly regulated (20, 22). MucR binds directly to the *btaE* and *omp25d* promoters and strongly represses the expression of these genes (6, 11, 20). VjbR-, MdrA-, and HutC-binding sites overlap the MucR binding site in the *btaE* promoter in *B. abortus* 2308 (11, 38, 39), and MdrA can displace MucR from this promoter in an electrophoretic mobility shift assay (11). Similarly, a predicted NolR-binding site overlaps the MucR binding site in the *omp25d* promoter in *B. abortus* (11, 37). These experimental findings suggest that MucR and antagonistic counter-silencers can work together to coordinate the proper temporal expression of adhesins in *Brucella* in response to host-specific environmental stimuli (Fig. 2A and B) in much the same fashion that H-NS/counter-silencer pairs coordinate the temporal expression of adhesin genes in other pathogenic bacteria such as *Yersinia enterocolitica* (44) and *Escherichia coli* O157:H7 (27). It is also interesting to note that the gene encoding the unipolar autotransporter adhesin BmaC, which like BtaE is important for attachment to epithelial cells and is produced at low levels during routine *in vitro* cultivation of *Brucella* cells (45), is strongly repressed by MucR in *B. abortus* and has binding sites for the cell cycle regulator CtrA that overlap MucR binding sites in its promoter (11, 32). This suggests that MucR and CtrA may work in concert to coordinate *bmaC* expression in response to cell cycle signals (Fig. 2C), which would be consistent with the observation that *Brucella* cells in G1 of the cell cycle are more invasive for mammalian cells than those in other phases of the cell cycle (33).

## QUORUM SENSING

**Fig 3:**
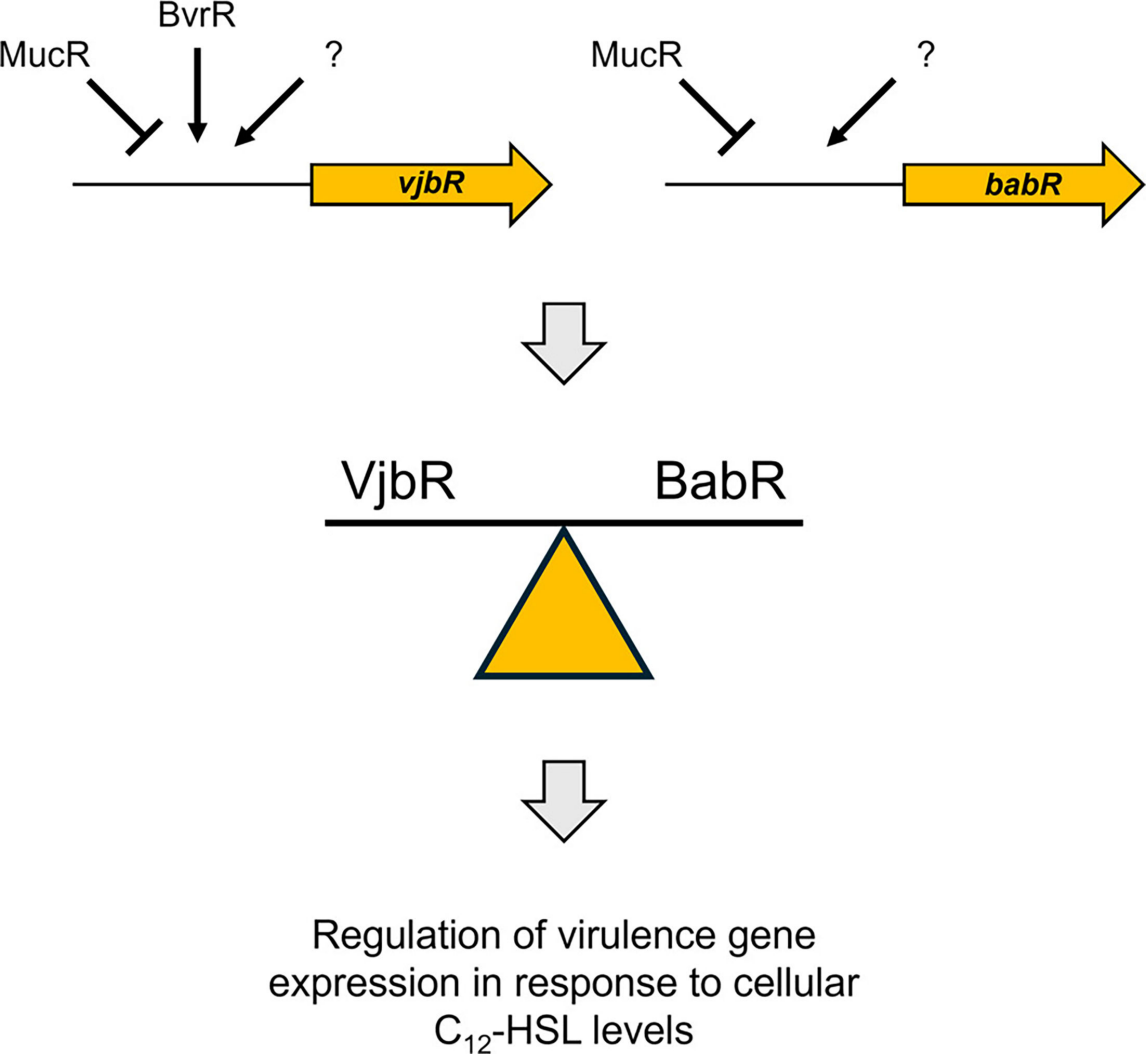
Proposed role of MucR in balancing the cellular levels of the *Brucella* quorum-sensing regulators VjbR and BabR. MucR and transcriptional regulators (known and unknown) regulate the expression of *vjbR* and *babR* (top), which affects the relative cellular levels of VjbR and BabR (middle) that ultimately modulate virulence gene expression in response to cellular C_12_-HSL levels (bottom).

 Two LuxR-type regulators, VjbR and BabR (aka BlxR), control virulence gene expression in *Brucella* in response to cellular C_12_-HSL levels (28–30, 46, 47). These two regulators control the expression of many of the same genes, but experimental infections in mice and cultured mammalian cells have shown that VjbR has a more dramatic effect on virulence than BabR. MucR binds directly to the *babR* promoter in *B. abortus* 2308 and *babR* expression is elevated >20-fold in an isogenic *mucR* mutant compared to 2308 (6, 11). Elevated *babR* expression has also been reported in *B. melitensis*, *B. ovis*, and *B. canis mucR* mutants (7, 19, 20). As shown in Table 1, there is also evidence suggesting that *vjbR* expression may be coordinately regulated by MucR and BvrR (11). This raises the possibility that MucR regulation of *babR* and *vjbR* expression may play an important role in balancing the cellular levels of these two quorum-sensing regulators and influencing their differential impacts on virulence gene expression (Fig. 3).

## TYPE IV SECRETION

 Multiple lines of evidence suggest that MucR regulates the genes encoding the Type IV secretion system in *Brucella* (7, 19, 20, 48). ChIP-seq and EMSA analysis have also demonstrated a strong binding affinity for MucR in the intergenic space between *virB1* and *virB2* in *B. abortus* (11) suggesting that MucR directly regulates these genes. However, as discussed in the previous section, MucR has a strong repressive effect on *babR* expression, and BabR regulates *virB* expression in *B. melitensis* (28, 47). Moreover, as shown in Table 1, evidence suggests that MucR and the transcriptional activators VjbR and BvrR may work in concert to coordinate the expression of the genes encoding the T4SS and its effectors in response to host-specific stimuli (11). This complex regulatory network could be envisioned to provide the brucellae with a mechanism for maximizing the expression of the T4SS genes at specific stages of infection when these genes provide their fitness benefits in much the same way that H-NS and H-NS-like proteins work in concert with antagonistic counter-silencers to coordinate the expression of the genes encoding the Type III secretion systems and their effectors in *Salmonella* (49) and *Pseudomonas* (50). But whether the MucR regulatory links to *virB* are essential for the wild-type virulence of *Brucella* strains remains to be experimentally determined.

## MucR AND THE EVOLUTION OF *BRUCELLA* VIRULENCE

**Fig 4:**
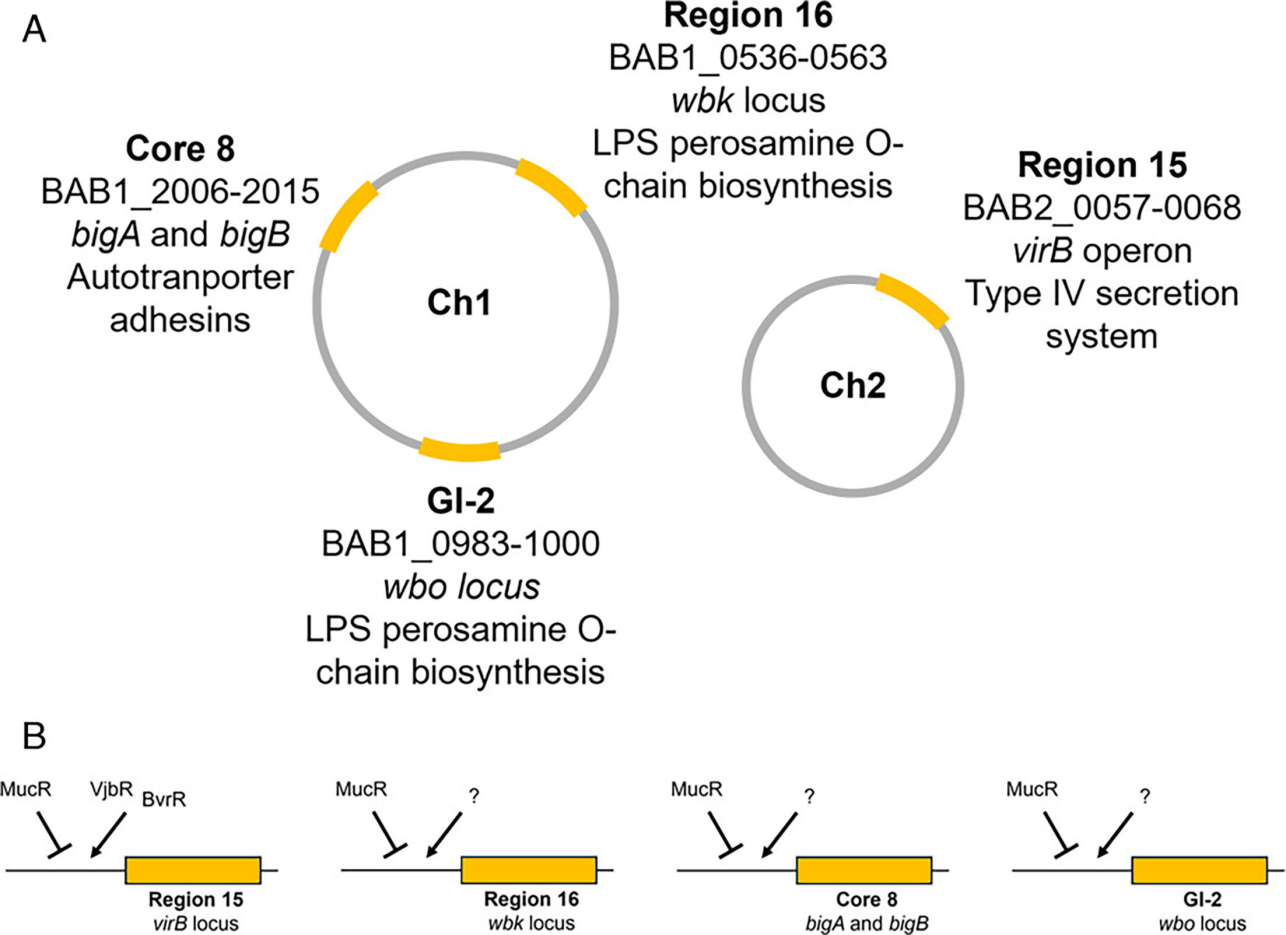
**(A)** *B. abortus* 2308 genomic islands encoding known virulence determinants that are bound by MucR (11) including Region 16 (52), GI-2 (53), and Core 8 (52, 54) on Chromosome 1 that encode the *wbk* locus, *wbo* locus, and *bigA/bigB*, respectively, as well as Region 15 (52) on Chromosome 2 that encodes the *virB* operon (**B**) Model for regulation of known virulence determinants encoded on genomic islands by MucR and other transcriptional activators.

 Like its H-NS and H-NS-like counterparts, MucR has been proposed to function as a xenogeneic silencer in the α-proteobacteria preventing the potentially toxic expression of genes acquired by HGT (51). Based on their intracellular niche and close association with their mammalian hosts, it is thought that the “classical” *Brucella* strains such as *B. melitensis*, *B. suis*, *B. abortus*, *B. canis*, and *B. ovis* have little opportunity to acquire novel genes by HGT (52). But it has been proposed that the acquisition of specific virulence genes such as those encoding the perosamine O-chain of the LPS, the T4SS, and the adhesins BigA and BigB from other bacteria by HGT played key roles in the evolution of these *Brucella* strains to become highly host-adapted mammalian pathogens (52–54), though recent examination of atypical *Brucella* spp. suggests that the acquisition, distribution, and role of HGT loci in the evolution of *Brucella* may be more complex (55). Consequently, it is interesting that the multi-gene genomic islands that encode the T4SS, LPS O-chain, and BigA and BigB adhesins in *B. abortus* 2308 are covered by broad MucR ChIP signatures (Fig. 4A) (11). Identifying the transcriptional activators that regulate the expression of these genes, defining the environmental stimuli that these regulators respond to, and determining how MucR and these regulators interact to coordinate the expression of these horizontally-acquired virulence genes (Fig. 4B) will give us a better picture of how MucR’s proposed role as a xenogeneic silencer has influenced the evolution of *Brucella* strains as mammalian pathogens.

## REGULATION OF MucR ACTIVITY

One aspect of the role of MucR in *Brucella* and related members of the α-proteobacteria that remains elusive is how the activity of MucR is regulated outside of interactions with functional transcriptional counter-silencers. While the transcription of *mucR* has been shown to be autoregulated in *Ensifer* (56), *Agrobacterium* (57), and *Brucella* (6), the effects of this regulation on MucR protein levels have not been thoroughly examined. In systems encoding H-NS and other H-NS-like proteins, post-translational mechanisms—including covalent modifications (e.g., phosphorylation and acetylation), targeted proteolysis, and interactions with binding partners and small molecules—regulate protein function and stability (58, 59). However, it is unclear whether similar regulatory mechanisms exist for MucR. In *E. loti*, one study failed to detect post-translational modifications of native MucR (60), but this was only characterized under a single growth condition and to our knowledge, post-translational modifications of MucR have not been examined in other members of the α-proteobacteria, including *Brucella* spp. Future experiments aimed at understanding the regulation of MucR activity will be important for understanding the complete role of MucR in the α-proteobacteria and *Brucella* virulence.

## CONCLUSIONS

** **MucR’s function as a nucleoid-structuring protein, its impact on the expression of a broad array of genes, and the pleiotropic fitness defects displayed by *Brucella mucR* mutants undoubtedly play important roles in the dramatic attenuation displayed by *mucR* mutant strains (6, 7, 11, 19, 20). However, as detailed in previous sections of this manuscript, evidence also suggests that the capacity of MucR to function as a gene silencer allows this protein to work in concert with antagonistic counter-silencers to ensure that specific virulence-associated genes are only highly expressed when their gene products provide fitness benefits during the infectious life cycle (11). Preventing the gratuitous expression of these genes would minimize wasteful energy expenditure and, in theory, prevent unproductive interactions of the brucellae with specific host tissues. This proposed function is consistent with the role that H-NS and H-NS-like proteins play in coordinating the proper temporal expression of virulence genes in other bacterial pathogens (25, 61, 62). Moreover, from an evolutionary perspective, MucR has been proposed to play a similar role in coordinating the proper temporal expression of “host-interaction” genes in plant pathogens and symbionts that are close phylogenetic relatives of the brucellae (5, 13). Future studies will tell us just how important these functions are in MucR’s role as an essential virulence determinant in *Brucella*. Additionally, further elucidation of how MucR oligomerization influences its interaction with DNA will be critical, as emerging structural data suggest that these parameters may modulate MucR function in ways distinct from H-NS and other H-NS-like proteins (12).
